# Osteogenic Differentiation Potential of Adipose-Derived Mesenchymal Stem Cells Cultured on Magnesium Oxide/Polycaprolactone Nanofibrous Scaffolds for Improving Bone Tissue Reconstruction

**DOI:** 10.34172/apb.2022.015

**Published:** 2020-09-22

**Authors:** Zahra Niknam, Ali Golchin, Mostafa Rezaei –Tavirani, Parviz Ranjbarvan, Hakimeh Zali, Meisam Omidi, Vahid Mansouri

**Affiliations:** ^1^Faculty of Paramedical Sciences, Shahid Beheshti University of Medical Sciences, Tehran, Iran.; ^2^Proteomics research center, Shahid Beheshti University of Medical Sciences, Tehran, Iran.; ^3^Department of Clinical Biochemistry and Applied Cell Sciences, School of Medicine, Urmia University of Medical Sciences, Urmia, Iran.; ^4^Medical Nanotechnology and Tissue Engineering Research Center, School of Advanced Technologies in Medicine, Shahid Beheshti University of Medical Sciences, Tehran, Iran.; ^5^Marquette University School of Dentistry, Milwaukee, WI, USA.; ^6^Protein Research Centre, Shahid Beheshti University, GC, Velenjak, Tehran, Iran.

**Keywords:** Adipose-derived stem cells, Electrospinning, Magnesium oxide, Osteogenesis, Polycaprolactone, Tissue engineering

## Abstract

***Purpose:*** Recently, bone tissue engineering as a new strategy is used to repair and replace bone defects due to limitations in allograft and autograft methods. In this regard, we prepared nanofibrous scaffolds composed of polycaprolactone (PCL) and magnesium oxide (MgO) nanoparticles using the electrospinning technique for possible bone tissue engineering applications.

***Methods:*** The fabricated composites were characterized via scanning electron microscopy (SEM) imaging of scaffolds and seeded cells, water contact angle, DAPI staining, and MTT assay. Then osteogenic differentiation of adipose-derived mesenchymal stem cells cultured on this composite scaffold was determined by standard osteogenic marker tests, including alkaline phosphatase (ALP) activity, calcium deposition, and expression of osteogenic differentiation genes in the laboratory conditions.

***Results:*** The SEM analysis demonstrated that the diameter of nanofibers significantly decreased from 1029.25±209.349 µm to 537.83+0.140 nm, with the increase of MgO concentration to 2% (*P* < 0.05). Initial adhesion and proliferation of the adipose-derived mesenchymal stem cells on MgO/PCL scaffolds were significantly enhanced with the increasing of MgO concentration (*P* < 0.05). The 2% MgO/PCL nanofibrous scaffold showed significant increase in ALP activity (*P* < 0.05) and osteogenic-related gene expressions (Col1a1 and OPN) (*P* < 0.05) in compared to pure PCL and (0, 0.5 and 1%) MgO/PCL scaffolds.

***Conclusion:*** According to the results, it was demonstrated that MgO/PCL composite nanofibers have considerable osteoinductive potential, and taking together adipose-derived mesenchymal stem cells-MgO/PCL composite nanofibers can be a proper bio-implant to usage for bone regenerative medicine applications. Future in vivo studies are needed to determine this composite therapeutic potential.

## Introduction

Bone tissue engineering is a new and developing option that can be the most effective substitute for the traditional invasive methods of bone replacement, including allografts and autografts.^1^ Around 4 000 000 bone grafting is performed worldwide annually, and bone grafts are the second most common transplanting tissue in medical surgeries.^2-4^ Both autografts and allografts possess excellent osteoinductive and osteoconductive properties. Still, they have limitations such as procurement morbidity and constraints on available quantities, limited source of the allografts, minor immunogenic rejection, and risk of disease transmission are unresolved issues.^5,6^ Therefore, the fabrication of scaffolds witch possesses biocompatibility, biodegradability, mechanical, and architecture properties similar to native bone tissue are very important in regenerative medicine researches.^7-9^ Hybrid composites can contribute conditions similar to the biological environment that the aggregate of composite compounds and stem cells from suitable sources provide natural niche for proliferation, differentiation, and cell viability. For instance, recently reported that white Portland cement enriched with ZnO and ZrO_2_ increased alkaline phosphatase (ALP) activity and Ca^2+^ ion release of human dental pulp stem cells over a defined time.^9^

Electrospinning is a suitable technique for the fabrication of continuous polymeric 2D and 3D scaffolds with varying diameters, ranging from nano to microscale.^10,11^ Nanofibrous scaffold witch created by electrospinning technique possesses a large surface-area-to-volume ratio, high specific surface, high porosity, various designs of surface functionality, and excellent mechanical properties.^12^ The most important feature of these scaffolds is to mimic the structural identity of the native extracellular matrix (ECM) in living tissues.^13^

Polycaprolactone (PCL) is a biodegradable synthetic polymer that has received FDA approval for biomedical applications. It is a relatively slow degrading polymer as well as has low melting temperature.^14^ There has been considerable attention in the use of electrospun PCL nanofibers as a proper scaffold for bone tissue engineering due to its nontoxic, semi-crystalline, biocompatible, bioresorbable properties as well as a low-cost synthetic polymer.^14^ However, PCL nanofibers have poor mechanical properties and show poor adhesion and proliferation of cells due to its hydrophobic surface and poor wettability.^15^ Some modifications and incorporation of other materials in the PCL scaffold can accommodate its deficiencies.^16^ For instance, with the addition of silicate-containing hydroxyapatite micro-particles to the PCL, a hybrid scaffold was obtained, which has shown randomly oriented and well-aligned microfibers, enhanced cell adhesion and viability during *in vitro*tests.^17^ Heydari et al reported that the electrospun scaffolds composed of PCL and octacalcium phosphate particles exhibited suitable mechanical properties and a positive impact on the growth of the osteoblast human G-292 cells on them.^18^ Besides, the surface of PCL nanofibers can be treated through hydrolysis using sodium hydroxide (NaOH) to improve the wettability, cell attachment, and proliferation on the scaffolds.^19-21^ Yeo et al reported that pretreatment of PCL scaffolds with NaOH could be increased the hydrophilicity and surface area for higher proliferation rate and differentiation level.^22^

Recently, the researchers reported that the composition of the polymer matrix with metals could modify their mechanical characteristics.^23^ Magnesium (Mg) is an essential element in bone tissue and ECM because it is mainly related to the cell differentiation, mineralization of calcined tissues, and indirectly influences mineral metabolism.^24-26^ Currently, Mg alloys were being considered for use as implant material due to their good biocompatibility, biodegradability, and satisfactory mechanical properties. Moreover, previous studies showed that Mg could directly enhance cell adhesion and proliferation, as well as osteogenic differentiation.^6,27-29^ For example, Hickey et al investigated the influence of incorporating magnesium oxide (MgO) nanoparticles in poly (l-lactic acid) (PLLA) and hydroxyapatite-PLLA composites for orthopedic tissue engineering applications.^30^ They demonstrated that MgO nanoparticles significantly increased attachment and proliferation of osteoblast on hydroxyapatite-PLLA nanocomposites. They also showed that these nanocomposite materials have suitable mechanical properties for cancellous bone applications. Wang, et al fabricated a Mg ion-implanted micro/nanostructured titanium surfaces using a Mg plasma immersion ion-implantation method.^31^ In their study, rat bone marrow mesenchymal stem cells (MSCs) were used for evaluating the cellular responses. They demonstrated a significant increase in the spreading and growth of these cells on the surfaces of Mg-implanted micro/nanostructures compared in control plates. As well as the expression of osteogenic marker genes including ALP, osteocalcin (OCN), and osteopontin (OPN) were upregulated on both surfaces. The osteoblastic differentiation of stem cells was enhanced with increasing concentrations of Mg ions.^29^

There are many benefits in using MSCs for differentiation, in comparison with other stem cell sources,^32^ for instance, (a) MSCs are readily accessible and can be isolated from a different adult and fetal tissues such as bone marrow, adipose tissues, umbilical cord, fetal liver, muscle, etc.; (b) They have the suitable potential for being differentiated in a variety of cell/tissue types, including adipocytes, osteoblasts, chondrocytes, myoblasts, etc.; (c) They can efficiently expand to clinical volume in a relatively short period; (d) MSCs can be processed and stored for a sufficient time for therapeutic plans, and (e) Several clinical trials of MSCs are available.^32^

**Figure 1:**
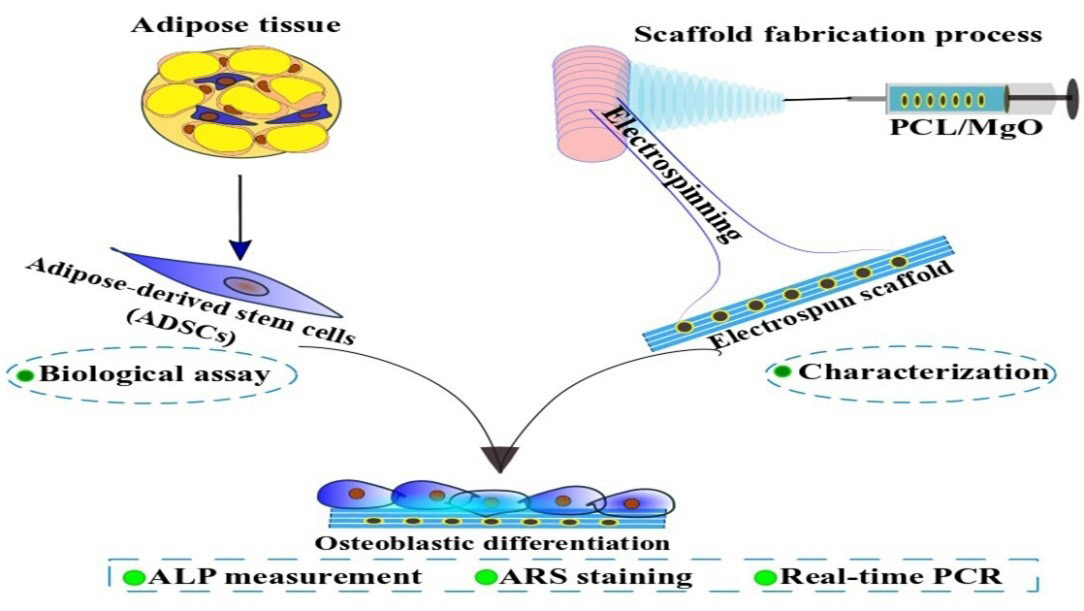
Scheme illustrating the experimental procedures of the current study.

In this study, MgO nanoparticles as a biodegradable metallic material and essential to bone development,^33,34^ are chosen to incorporate into PCL nanofibers. This study was designed to evaluate the in vitro biological response of electrospun MgO/PCL scaffolds, including adhesion, viability, and osteogenic differentiation of rat adipose-derived mesenchymal stem cells (ADSCs) (Figure 1). Therefore, the electrospun scaffolds were fabricated and characterized via scanning electron microscopy (SEM), water contact angle, and also biological behavior tests include in SEM observations, DAPI staining, and cell viability (MTT test). Then osteogenic differentiation of ADSCs cultured on this composite scaffold was determined by standard osteogenic marker tests, including ALP activity, calcium (Ca) deposition (Alizarin Red S), and expression of osteogenic differentiation genes in the laboratory conditions.

## Materials and Methods

### Fabrication of electrospun MgO/PCL nanofibers

The MgO/PCL nanofibrous scaffolds were fabricated by electrospinning technique. The PCL (MW = 80 000, Sigma Aldrich, St. Louis, MO) granules were dissolved in DMF (Merck, Germany, Cat no: 616-001-00-X): CHCl_3_ (Merck-Germany, Cat no: K45116345347) (2:8) to obtain a 10% w/v solution and homogeneous mixture. Afterward, MgO powder was gradually added with the ratios of 1:0, 1:0.005, and 1:0.01. Each solution was separately mixed using a vortex for 30 min. The polymeric mixture was filled into a 10-mL plastic syringe with a 20-gauge stainless steel needle, and then electrospinning was carried out. The syringe tip was charged to a supplied high-voltage (18 kV), and the steady-state flow rate was set to 1 mL/h. The polymer solution was deposited as solid fibers onto a fixed grounded aluminum sheet at 10 cm from the needle tip. The collected electrospun nanofibers were subsequently vacuum-dried for at least 48 hours to remove any residual solvents.

### Surface modification of PCL scaffolds

The PCL nanofibers were soaked in NaOH solution (1 M; pH = 12) for 4 hours and shaken gently at room temperature. After that, the scaffolds were washed twice with distilled water to neutralize the mixture.

### Surface wettability measurement of electrospun scaffolds

For wettability measurements, static water contact angles were obtained using a CAM 200 optical angle goniometer at room temperature. Five droplets of water (10 µL) were deposited on the different independent locations on each sample, and after 5 minutes, the average values of the data were reported.

### Surface morphologies of the nanofibers

The surface morphology and diameter of PCL and MgO/PCL nanofibers were characterized using field-emission SEM at an accelerating voltage of 15 kV and via EDS at 200 kV (FE-SEM, S-4800, Hitachi). Before FESEM imaging, each sample was sputtered with gold using a sputter coater.

### Isolation and culture of ADSCs

Rats (Wistar albino, male, 250-300 g) were housed under standard conditions. After sacrifice, subcutaneous adipose tissues were excised from their inguinal. Adipose tissues were washed three times in 30 mL PBS (Sigma, USA, Cat no: 1002381421), minced with a scalpel, and digested for 1 hour at 37°C with an equal volume of type I collagenase (0.2%; Sigma, Lakewood, N.J, USA). The digested tissues were neutralized with an equal volume of high-content glucose Dulbecco’s Modified Eagle’s Medium (DMEM, Bioidea, Iran) containing 10% fetal bovine serum (FBS, Gibco, South America, Cat no: 10270-106) and 1% antibiotics (100 U/mL penicillin and 0.1 mg/mL streptomycin, Bioidea, Iran, Cat no: BI1036). The digested adipose tissue was centrifuged at 700 rpm for 3 minutes, and the cell pellet was passed through a 70-µm filter (Becton Dickinson, Franklin Lakes, N.J., and the USA) to eliminate debris and yield single-cell suspensions. ADSCs were seeded in 25-cm tissue culture dishes and cultured in complete growth media (containing DMEM, 10% FBS, and 100 U/mL penicillin and 0.1 mg/mL streptomycin) at 37^◦^C under 5% CO2. The media culture was changed every 3 days; after that, until the confluency of cells in the flasks was reached to 80-90% the cultured cells were passaged using 0.25% trypsin-EDTA solution (Bioidea, Iran, Cat no: Bi1033). All experimental procedures were performed according to the Animal Care Committee guidelines, and the experimental protocol was approved by the Ethics Committee of Shahid Beheshti University of Medical Sciences.

### Flow cytometry

After the 3^rd^ passage, cells (1×10^6^) were suspended in 500 μL PBS containing 5% FBS. Aliquots from this cell suspension were treated with monoclonal antibodies conjugated with fluorescein isothiocyanate, against surface markers [CD44, CD73, and CD90 (BD Biosciences, Palo Alto, CA, USA)] for 45 minutes at 4°C. After that, the cells were washed three times in PBS with 5% FBS and resuspended in 1 mL PBS for analysis. Cell fluorescence was evaluated in a *FACSCalibur* Cytometer (FC 500; Beckman Coulter, Fullerton, CA, USA). The data obtained were analyzed using FlowJo software.

### ADSCs morphology and adhesion studies on scaffolds

The SEM images and DAPI staining were used to evaluate the morphology and adhesion of ADSCs on nanofibrous scaffolds. The ADSCs (1× 10^4^ cell/cm) were seeded on each sample in a 48-well plate. The cells were cultured in complete growth media (containing DMEM, 10% FBS, and 100 U/mL penicillin and 0.1 mg/mL streptomycin) at 37°C under 5% CO_2_ for 1-2 days. For SEM images, the cell/scaffold constructs were washed with PBS, to remove debris and unattached cells, and fixed with glutaraldehyde solution (2.5%, Sigma, USA) for 1h at room temperature. After further washing steps with PBS to remove excess glutaraldehyde, they were dehydrated through washing with graded ethanol series. The dried scaffolds were coated with gold using a sputter-coater (JEOL, JFC 1600). For SEM viewing and cell morphology assessment, an accelerating voltage of 3 kV was used.

For DAPI staining, the attached cells on scaffolds were rinsed with PBS and fixed with 10% formalin for 30 min. After rinsing the samples with PBS buffer, the fixed cells were permeabilized for 5 minutes in 0.1% Triton X-100. The cell nuclei were stained with 0.1 µg/mL of DAPI solution for 10 min. Next, the cell-scaffold constructs were washed three times with PBS and investigated using a fluorescence microscope (Zeiss Axiovert 200; Carl Zeiss Inc., Thorn -wood, NY, USA).

### Cell viability of the MgO/PCL composites

The viability and proliferation of ADSCs on nanofibrous scaffolds were determined by 3-[4, 5-Dimethylthiazol-2-yl]-2, 5-Diphenyltetrazolium Bromide (MTT) (Alfa Aesar, Great Britain, Cat no: L11939) assay. The scaffolds were placed in a 96-well plate, and 1× 10^4^ cells/cm^2^ ADSCs were seeded on each sample. The cells were incubated in complete growth media (containing DMEM, 10% FBS and 100 U/mL penicillin and 0.1 mg/mL streptomycin, all Invitrogen) at 37°C under 5% CO2 for 1, 4 and 7 days. In this assay, the culture medium in the disks was aspirate, and cells/scaffold constructs washed with PBS, and 100 µL RPMI1640 (Bio-IDEL) was added to each well. Then, 10 µL of MTT solution (5 mg/mL) was added to the culture wells and incubated for 4h at 37°C and 5% CO_2_. The formed formazan, purple-colored crystals implies the presence of viable cells. The crystals were dissolved by adding 100 µL dimethyl sulfoxide (DMSO) (Merck- Germany, Cat no: K47112112036) to each well, incubating at 37°C and 5% CO_2_ for 10 min. Finally, the absorbance value was recorded by an ELIZA reader (URIT-660, China) at a visible absorption wavelength of 570 nm and a reference wavelength of 630 nm. The cell viability was determined by the optical densities.

### Alkaline phosphatase activity

The osteogenic differentiation potential of MgO/PCL scaffolds compared to pure PCL was determined by the ALP measurement. 1×10^5^ cells per ml ADSCs were seeded on PCL and MgO/PCL nanofibrous scaffolds as above in the presence of 50 μg/mL ascorbic acid, 100 nM dexamethasone and 10 mM beta-glycerophosphate. After incubating for 7 and 14 days, scaffolds were washed three times with PBS, and cells were lysed using 1×RIPA buffer. Ultrasonication of cell-seeded scaffolds was carried out to agitate and disrupt the cell membranes. After centrifugation at 12 000 g for 10 minutes at 4°C, the supernatant fractions were collected and incubated with p-nitrophenyl phosphate solution for 30 minutes at 37°C. Reactions were stopped with 500 μL 1 N NaOH. The absorbance of the reaction product was determined using a microplate reader at 405 nm. For Quantitative analysis, ALP activity was normalized by total protein concentration. Bradford protein assay reagent (Thermo Fisher Scientific) was used for the measurement of entire protein content. The obtained results were expressed as U/mg protein.

### Alizarin Red S (ARS) staining assay

The ADSCs were seeded on nanofibrous scaffolds with different concentrations of MgO nanoparticles, as described earlier. After 21 days, the ARS staining assay was used to evaluate the calcium mineralization on samples. Briefly, after removing the media, the scaffolds were washed three times in PBS. The cells were fixed by immersion in 10% formalin for 30 minutes at room temperature, followed by washing with distilled water. Then, the cells were stained with ARS solution (40 mM, pH 4.2, Sigma) at room temperature for 30 minutes with gentle shaking. The plates were then washed thoroughly in distilled water, and samples were incubated with 10% cetylpyridinium chloride for 15 minutes. After that, to determine the levels of Ca deposit in the scaffolds, the absorption of supernatant solutions was measured at 405 nm.

### Real-time RT polymerase chain reaction (real-time RT-PCR)

After 7 and 14 days of culture of ADSCs on the PCL and PCL/MgO nanofibrous scaffolds in osteogenic medium, total ribonucleic acid (RNA) was extracted using Trizol reagent (YTA, Iran, Cat no: YT9063). Double-stranded cDNA was synthesized using 1 μg of total RNA with Thermo Scientific DyNAmo cDNA Synthesis Kit. Afterward, the real-time polymerase chain reaction (PCR) reaction was conducted by ABI PRISM 7500 sequence detection system (Applied Biosystems, USA, Cat no: 4368814) using the SYBR Green PCR Master Mix (BIOFACT, Korea, Cat no: DQ383-40H). PCR amplification was carried out under the following conditions: denaturation at 95°C for 5 seconds, followed annealing, and extension for 30 seconds at 56°C for 40 cycles. The relative mRNA expression levels of target genes were normalized to the glyceraldehyde-3-phosphate dehydrogenase as a reference gene and determined using the 2-^ΔΔCt^ method. In this research, the real-time PCR primers for specific genes, including runt-related transcription factor 2 (Runx-2), OPN and collagen type I (Col1a1), as osteogenic markers, were used (Table 1).

**Table 1:** Primer pair sequences

**Gene Forward primer Reverse primer**
Gapdh 5ʹ-GATGGTGAAGGTCGGAGTG-3ʹ 5ʹ-TGTAGTGGAGGTCAATGAATGG-3ʹ
Col1a1 5ʹ-CTCGCTCACCACCTTCTC-3ʹ 5ʹ-TAACAACTGCTCCACTCTG-3ʹ
Runx2 5ʹ-AGGAATGCGCCCTAAATCACT-3ʹ 5ʹ-ACCCAGAAGGCACAGACAGAAG-3ʹ
Opn 5ʹ-TCTGATGAGACCGTCACTGC-3ʹ 5ʹ-AGGTCCTCATCTGTGGCATC-3ʹ

### Statistical analysis

All analyses were performed using SPSS software. *P* values < 0.05 were considered as statistically significant. All data were reported as mean ± standard deviation (SD). Student’s *t* test was used to compare the results among different groups. Each experiment was repeated separately 3 times.

## Results and Discussion

In this study, MgO incorporated PCL nanofibers were successfully fabricated by the electrospinning technique. Electrospinning is a cost-effective, easy to use and versatile method to construct nanofibrous scaffolds for tissue engineering applications.^35^ Fabricated electrospun scaffolds were compared in terms of physical and biological properties to determine MgO potential for bone tissue regeneration and its biocompatibility.

### Characterization of electrospun MgO/PCL composite scaffolds

**Figure 2:**
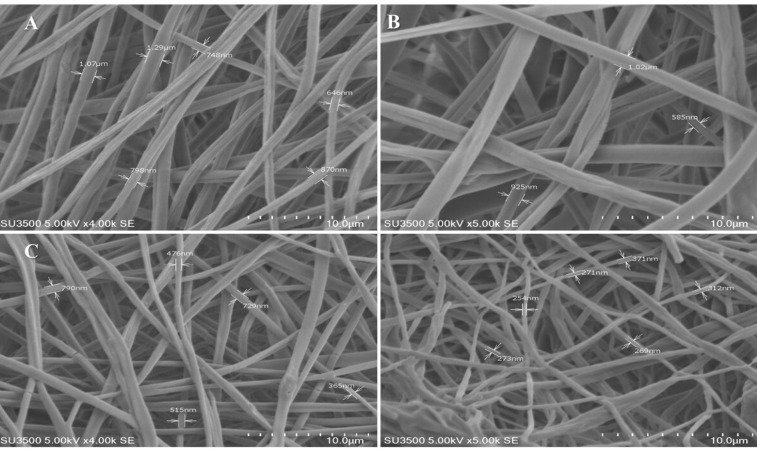
Surface morphological and constructional images of MgO/PCL composite nanofibers with different MgO concentrations (wt%): SEM images of (A) PCL, (B) MgO (0.5%)/PCL, (C) MgO (1%)/PCL, (D) MgO (2%)/PCL. (Scale bar = 10 μm).

**Figure 3:**
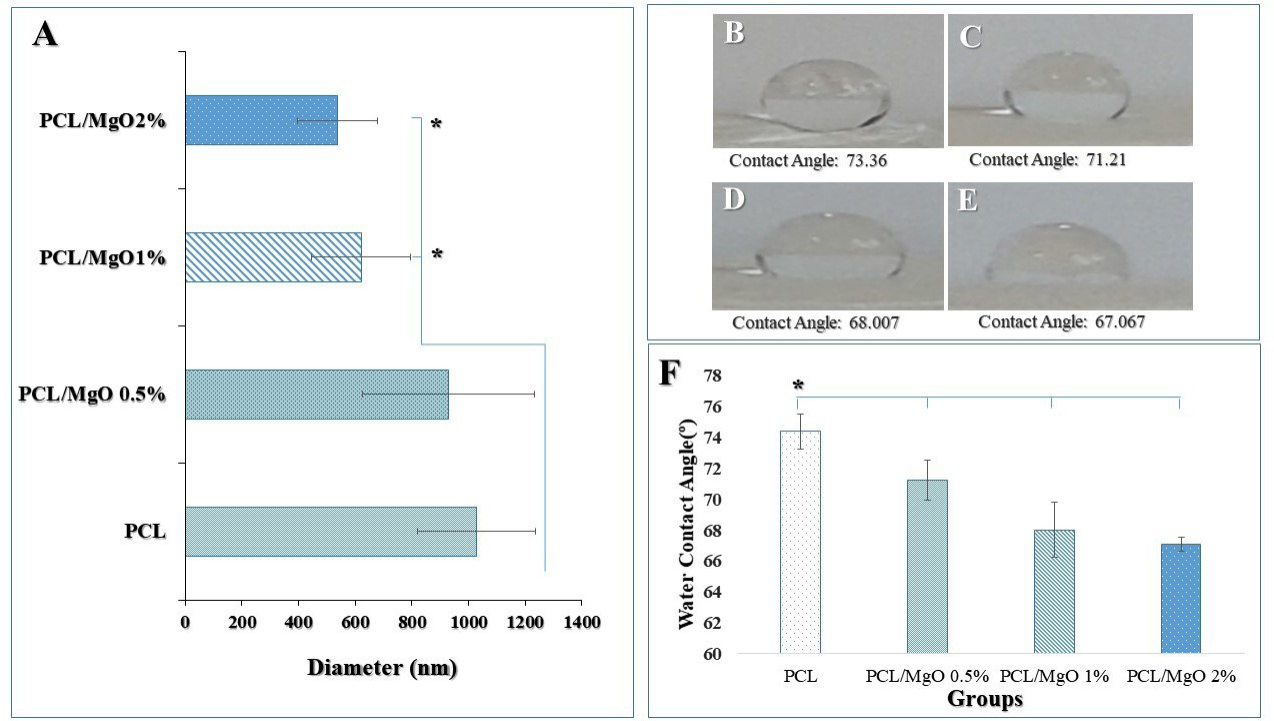
(A) The mean size of nanofibers in different samples (average of 20 nanofibers ± SD). There was a significant reduction in the size of 1 and 2 % MgO/PCL fibers compared to PCL fibers (*P < 0.05). Water droplets on various scaffolds after 5 min (B) PCL, (C) MgO (0.5%)/PCL, (D) MgO (1%)/PCL, (E) MgO (2%)/PCL. (F) Comparison of the water contact angle of the PCL and composites. When the MgO nanoparticle was incorporated in the PCL scaffold and its concentration in MgO/PCL composites increased, the water contact angle significantly decreased from 74.368˚ to 67.04˚ after 5 minutes (n=3) (T-tests) (*P < 0.05).

Electrospun PCL scaffolds have low cell attachment, ALP activity and mineralization,^36^ so MgO was incorporated into PCL nanofibrous scaffolds to accelerate or enhance osteogenic differentiation of ADSCs for subsequent bone engineering applications.^37^ Solutions of MgO/PCL in percent of 0.5%, 1%, and 2% were prepared and yielded nanofibers. Figure 2 shows SEM images of pure PCL^6^ and composite scaffolds. Morphology studies of nanofibers illustrated that the fibers were randomly oriented, and possessed interconnected and nanoscale pores. The average diameter of the pure PCL nanofibers significantly was more than the other fabricated test nanofibers (Figure 3A). As seen in Figure 3A a decreasing trend in the thickness of the PCL nanofibers was observed from 1029.25±209.349 µm to 537.83+0.140 nm with the increasing of MgO concentration to 2%. It seems that the conductivity of the filler-polymer solution is enhanced during increasing the Mg and oxygen ions, and consequently, nanofibers diameter is reduced.^38,39^ Kim et al investigated the effects of adding different concentrations of ionic salts to distilled water to prepare a solution of Poly (2-acrylamido-2-methyl-1-propane sulfonic acid) for electrospun nanofibers fabrication.^40^ They observed the decreasing fiber diameter with increasing conductivity of the solution since the fiber jet produced from a high conductive solution is exposed to a greater stretching effect when influenced by an applied voltage. It has also been proved that the radius of the electrospinning jet is inversely related to the cube root of the electrical conductivity of the solution.^41^ Besides due to the presence of filler nanoparticles in nanofibers, as shown in Figure 2 the surface morphology of the fibers in MgO/PCL scaffolds became rough, which may be useful for enhancing the biological function of scaffolds and improve cell attachment.^42,43^

In general blend-composite systems with synthetic and natural polymers have a high hydrophilic property than the pure synthetic polymers, and this is because of the presence of different functional groups in natural materials.^44^ Hydrophilic properties of scaffolds are most prominent for cell attachment, proliferation and so differentiation as well.^45^ In this research, it is expected to the hydrophilicity of scaffolds increases due to the incorporating of MgO nanoparticles in PCL synthetic polymer and surface modification of scaffolds using NaOH solution. The wettability of fabricated composite scaffolds was determined by measuring the contact angle. Figure 3 (B, C, D, E) shows the water contact angle of pure PCL scaffold and the MgO/PCL composites. As shown in Figure 3F, the PCL scaffold indicated an approximately higher water contact angle rather than MgO/PCL scaffolds. It can be acceptable because of hydrophobic properties and a low rate of water absorption in the PCL scaffold.

However, when the MgO nanoparticle is incorporated in PCL scaffolds and its concentration in MgO/PCL composites increases, the water contact angle decreases from 74.368° to 67.04° after 5 minutes. MgO intrinsically is hydrophilic,^46^ that influences the adsorption of serum proteins.^38^ As well as nanoscale roughness has been shown to influence surface wettability,^47^ it is estimated that MgO nanoparticles with enhancing the surface roughness of PCL nanofibers, provided more hydrophilic surfaces. The obtained results of this study also showed that MgO nanoparticles enhance the hydrophilic properties of the PCL electrospun scaffold. Recently yang et al showed that by modification of the macroporous PLLA scaffolds, under optimal treating conditions, using a mixture of aqueous 0.25-M NaOH and ethanol, the surface properties of PLLA, such as hydrophilicity and roughness, were significantly increased.^48^ Besides, the affinity, attachment, and growth of seeded cells on the modified surface were considerably increased.^48^ Also, in our study, the fabricated scaffolds hydrolyzed by 1M NaOH solution due to increasing their biocompatibility. By hydrolysis, the ester bonds of PCL are cleaved and lead to the carboxylic acid and hydroxyl groups that are exposed to the polymer surface.^49^ This modification yielded significant improvements in hydrophilicity, initial cell attachment, and protein adsorption.^22^

### Cell behavior on scaffolds

**Figure 4:**
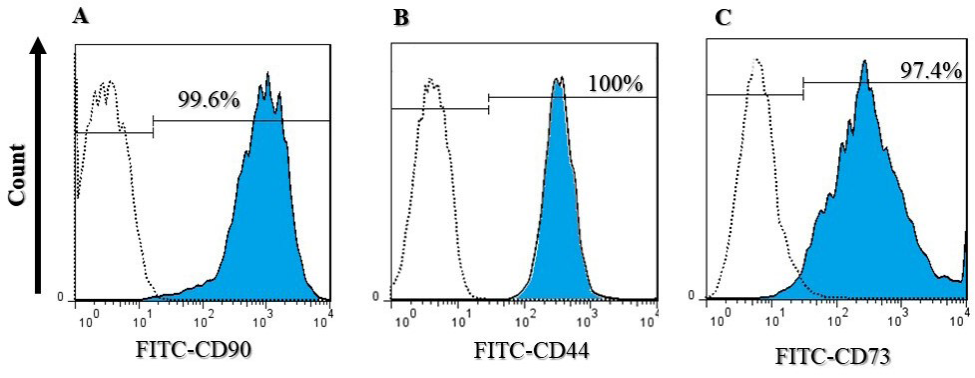
Flow cytometry analysis of ADSCs labeled with (A) CD90, (B) CD44, and (C) CD73 conjugated to FITC. Cells were 99.6% positive for CD90, 100% positive for CD44, and 97.4% positive for CD73.

Adipose-derived stem cells were confirmed using flow cytometry and analyzed cell surface markers. Cells were 99.6% positive for CD90, 100% positive for CD44, and 97.4% positive for CD73, which are all ADSC surface protein markers (Figure 4).^6^

**Figure 5:**
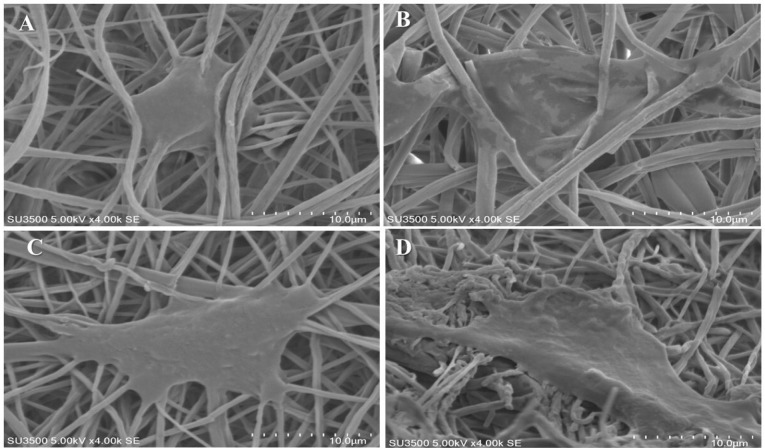
Morphology of ADSCs-seeded on MgO/PCL composite scaffolds with different MgO concentrations: SEM images of cell morphologies after 1 days of culture: (A) PCL, (B) MgO (0.5%)/PCL, (C) MgO (1%)/PCL, (D) MgO (2%)/PCL. (Scale bar = 10 μm).

**Figure 6:**
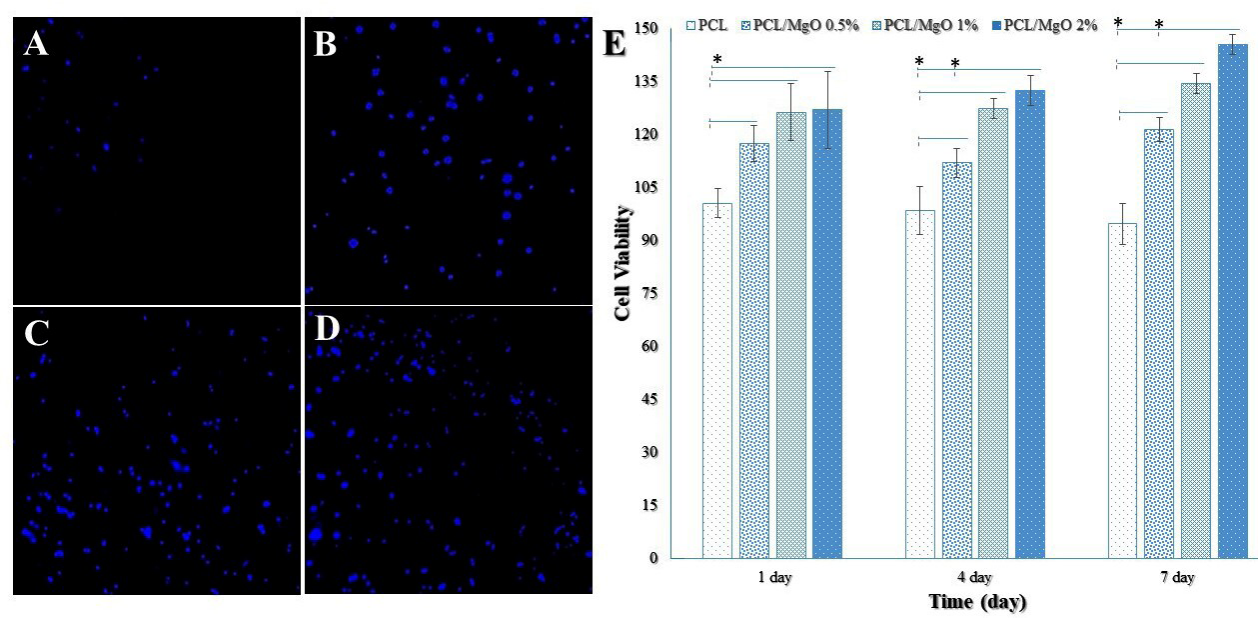
Fluorescence microscope images for DAPI staining of ADSCs cultured for one day on nanofibrous scaffolds. (A) PCL, (B) MgO (0.5%)/PCL, (C) MgO (1%)/PCL, and (D) MgO (2%)/PCL (Scale bar = 100 μm). (E) Cell viabilities of ADSCs on pure PCL and MgO/PCL composites using MTT assay. The absorbance was detected under a wavelength of 570 nm and the reference wavelength of 640 nm to determine the cell viability in comparison to the control (PCL only). The cell viability and proliferation of ADSCs was significantly higher in the MgO/PCL composites compared to the pure PCL at 1, 4, and 7 days. As well as the proliferation rates of ADSCs gradually increased with enhancing the concentration of MgO nanoparticles with the extended culture time (n=3) (*t*tests) (*P < 0.05).

The initial behaviors of cell attachment and proliferation are critical to revealing the cell-scaffold interactions that profoundly affect cellular differentiation.^50^ Physicochemical properties of MgO/PCL scaffolds such as the diameter of nanofibers, hydrophilicity, and surface roughness, may facilitate cell adhesion and proliferation.^12^ Several studies reported that even low concentration of Mg might have an insulin-like effect on cell proliferation,^51,52^ which has been observed in our study. The efficiency of the scaffolds in supporting the ADSCs was assessed by SEM images (Figure 5) and DAPI staining (Figure 6A, B, C, D). Figures 5 and 6 show the stained cells by DAPI and SEM images of seeded ADSCs on PCL and MgO/PCL scaffolds after one day. Our results demonstrated that ADSCs were successfully attached to the surfaces of the scaffolds. Simultaneously, it could be seen that with increasing MgO concentration in composite scaffolds, ADSCs indicated increasing in spreading with many filopodia and flattened with a more significant attachment in comparison to the cells on pure PCL nanofibers. It is thought that reducing the diameter of nanofibers in composite scaffolds could be a factor in better adhesion and further expansion of cells on the surface of scaffolds. In recent years, previous studies have indicated that the diameter of the electrospun fiber has a significant effect on cell behavior such as cell-spreading, proliferation, differentiation, and gene expression.^53,54^ In one study, fibers with a diameter of 0.347 μm, 0.947 μm, and 6.48 μm were fabricated by electrospinning for evaluating their effect on MC3T3-E1 pre-osteoblasts behavior.^55^ Their results showed that the cells seeded on 0.35 μm scaffolds demonstrated a higher projected area and upregulation of osteogenic gene markers, including Runx2, Col I, ALP, and OCN. A higher proliferation rate and aspect ratio were observed for cells seeded on 6.5 μm scaffolds. Chen et al^56^ assessed how the specific surface area of electrospun scaffolds and fibers diameter influenced cell adhesion and spreading. They showed that nanofibrous scaffolds enhanced cellular attachment compared to microfibrous scaffolds. Considerably, in scaffolds with specific surface area <7.13 microm (-1), cell attachment rate stayed mainly unchanged; however, for specific surface area > 7.13 microm (-1) and with an increase in its amount, significant enhancement in cellular adhesion rate was shown. These results demonstrated that decrease in fibers diameter provide a faster cell attachment in compared to larger diameters. Also in this research as mentioned above, with increase in MgO concentration in the PCL nanofibers, specifically in MgO (2%)/PCL with lower fibers diameter, the ADSCs illustrated significantly adhesive, spreading, mature, and scattered focal adhesions, which indicates a strong interaction between the cells and the surface of the scaffold.

On the other hand, several studies have indicated that cell attachment, proliferation, and orientation affected by morphology and roughness of electrospun fibers.^57-61^ Zareidoost et al reported that the culture of osteoblast cells on treated different groups of titanium surfaces to evaluate the cellular response.^62^ They demonstrated that surfaces with higher roughness had better biocompatibility compared to controls. They concluded that surface roughness of different groups of titanium is correlated to osteoblastic cell attachment, viability, and morphology in comparison with smooth titanium. Cells can sense topography of materials through the filopodia that are rod-like cell surface protrusions contain bundles of parallel actin filaments. Filopodia enable orients and spread the cell along the direction of the nanoscale features presented onto a nanofibrous surface.^63^ Therefore in our study with the incorporation of MgO nanoparticle in the PCL nanofibers and with increasing its concentration, it seems the surface of MgO/PCL composite nanofibers became rougher and more irregular, as well as increased hydrophilicity, which exhibits filopodia on the cell surface and improves cell attachment, spreading and growth.^12,38,64^ Moreover the DAPI staining of ADSCs revealed poor cell adhesion on PCL nanofibers, whereas the entire volume of the MgO/PCL scaffolds was covered with cells demonstrating better adhesion on the composite nanofibrous scaffolds. It can be concluded that the presence of MgO nanoparticles in nanofibrous scaffolds can significantly promote the initial cell attachment and spreading.

Figure 6E shows the ADSCs viability on the pure PCL and MgO/PCL composites. The cell viability was significantly higher (*P* < 0.05) in the MgO/PCL composites compared to the pure PCL at 1, 4, and 7 days, as well as the proliferation of ADSCs gradually increased with enhancing the concentration of MgO nanoparticles with the extended culture time. Cell viability on the pure PCL scaffold was decreased over time, which it seems according to its hydrophobic surface. Significant differences were also seen in cell viabilities between ADSCs cultured on MgO (0.5%)/PCL scaffolds and ADSCs cultured on MgO (2%)/PCL at 4 and 7 days. The proliferation rates of ADSCs on the MgO (1%)/PCL and MgO (2%)/PCL composite scaffolds did not significantly differ. These results indicated that with increasing MgO concentration, the biocompatibility of scaffolds and cell viability increased. According to recent studies, except hydrophobicity, PCL might promote favorable cellular activities and support cell attachment, proliferation, and differentiation.^65,66^ Nonetheless, our studies of cell adhesion and viability indicated that MgO incorporated into PCL scaffolds could speed up the adhesion, viability, and proliferation of ADSCs on MgO/PCL scaffolds. Kazimierczak et al^67^ synthesized nano-hydroxyapatite substituted with Mg^2+^ ions for fabricating chitosan-agarose-hydroxyapatite scaffolds with superior biocompatibility. They found that incorporation of Mg^2+^ ions into fabricated structure significantly enhanced the spreading of the cells and promoted cell proliferation on the scaffold surface, which provided more robust support for our results. It is possible because of the hydrophilic feature of the MgO, porous structure, high surface roughness, and high surface area of electrospun nanofibrous scaffolds that created a good environment for cell adhesion and proliferation.^68^ On the other hand, these results could be possible owing to the positive effect of releasing of Mg^2+^ ions in the local condition. It is estimated that the stimulatory effects of free Mg^2+^ ions be through two possible mechanisms: (i) activation of the integrin family present on the cell membrane, (ii) gap junctional communication.^38^ Initiate conformational of membrane-bound integrin α-chain adhesion receptors can be changed due to the binding of Mg^2+^ ions to sites on them. That way, Mg^2+^ ions seem to contribute to the activation of the integrin family present on the cell membrane for ligand binding and helping to cells attachment to biomaterials and composite scaffold surfaces. Consequently owing to improve cell adhesion, increase cell proliferation, differentiation, and eventually ossification.^38,69^ He et al^70^ reported that cell viability, alkaline phosphate activity, and OCN expression of human osteoblasts were significantly increased in the presence of Mg ions. As well as they showed gap junction intercellular communication of osteoblasts was promoted considerably by Mg ions. Gap junction intercellular communication mediated by connexins, in particular connexin 43 (Cx43), promote transmitting signals and nutrient molecules between bone cells, which are responsible for bone development and remodeling. Besides, gap junction intercellular communication transmits anabolic effects of growth factors and hormones, broadcasts Ca^2+^ signaling, and regulates expression of osteoblast differentiation genes and markers.^70^ It is hypothesized that Mg ions can promote the activity of osteoblasts through increasing gap junction intercellular communication between cells, and effect on bone formation.^38^

### In vitro osteogenic differentiation

**Figure 7:**
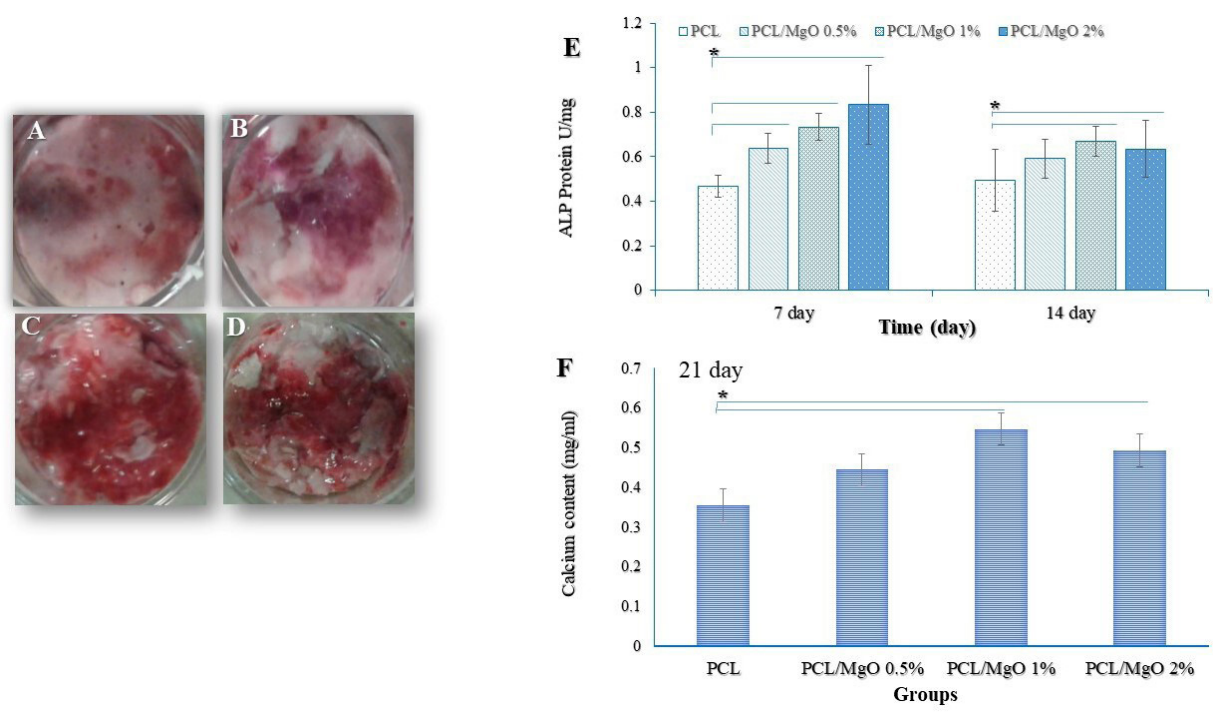
(A) ALP activity of ADSCs grown on PCL and MgO/PCL scaffolds. The absorbance was detected under a wavelength of 405 nm, and the ALP activity was normalized to the total protein level of the samples. ALP activity was significantly higher in cell culture of MgO/PCL scaffold groups compared to pure PCL scaffolds on days 7 and 14 (*P < 0.05). It is evident that, on the 7th day, with the addition of MgO concentration in composite scaffolds, the ALP activity of cells was gradually increased. Alizarin Red staining, an anthraquinone dye that has been widely used to evaluate calcium deposits in cell culture (C) PCL, (D) MgO (0.5%)/PCL, (E) MgO (1%)/PCL, (F) MgO (2%)/PCL. (B) Calcium deposition of ADSCs grown on PCL and composite scaffolds after 21 days. There were significant increases in calcium content in MgO (1%)/PCL and MgO (2%)/PCL composite scaffolds in comparison whit PCL fibers (n=3) (*t*tests) (*P < 0.05).

The stages of bone development are described by three phases: proliferation, ECM maturation, and mineralization, which are determined by the expression of special osteoblastic markers.^71^ Among the important osteogenic hallmarks, the up-regulation of ALP activity is a fundamental event occurring at the early time points of osteoblastic differentiation and immature osteoblast activity.^72,73^ ALP helps the secretion of ECM for deposition before mineralization. ALP cleaves organic phosphate esters and has a leading role in the formation of apatite Ca phosphate.^74^ Figure 7A indicates the in vitro ALP activity of ADSCs cultured on MgO/PCL scaffolds at 7 and 14 days. ALP activity was significantly higher in cell culture of MgO/PCL scaffold groups compared to pure PCL scaffolds on days 7 and 14 (*P* < 0.05). It is evident that, on the 7th day, with the addition of MgO concentration in composite scaffolds, the ALP activity of cells was gradually increased. It seems in our study that MgO is released from PCL scaffolds because MgO promotes osteoblast differentiation of ADSCs and ALP activity was increased in early time (7^th^ day). However, on the 14th day, ALP activity of ADSCs among MgO/PCL groups was not indicated a significant difference. In addition, ALP amount in ADSCs grown on three MgO/PCL scaffolds decreased compare to 7^th^ day, which can be related to the aforementioned fact that ALP activity is an early indicator of immature osteoblast activity. Moreover, reducing ALP activity on the 14^th^ day especially in 2% MgO/PCL scaffold compare to 7^th^ day can be due to entering the next cellular process such as switching to mineralization step at primary time, as reported in earlier studies.^12,75,76^ In one study also Suryavanshi et al reported that MgO-loaded electrospun PCL seeded with osteoblast-like MG-63 cells indicated the enhanced biological performance of cells, including adhesion and proliferation, as well as increased ALP activity because of nano topography, higher surface roughness, increased hydrophilicity and protein adsorption of composite scaffolds.^38^

After evaluating the ALP activity, the efficiency of the mineralization was analyzed as a late osteogenic differentiation marker, which calcifies.^77^ The ARS staining was used to illustrate the amount of Ca minerals,^78^ and the intensity levels of Alizarin red were quantitatively analyzed. As expected from ALP activity results, MgO was capable of raising the amount of matrix mineralization. Figure 7 (C, D, E, F) shows the mineralized matrix formed in ADSCs grown on samples. The amount of Ca deposited on ADSCs have grown on MgO/PCL composite scaffolds with 1- and 2-% MgO was significantly higher than the deposition on ADSCs have grown on pure PCL scaffolds (*P* < 0.05) (Figure 7B).

Moreover, cell adhesion, well spreading, and morphology of cells on scaffolds can influence intracellular responses and cell differentiation.^50^ As recent studies have shown, PCL scaffolds alone cannot provide good responses for cellular behaviors, which is one of the essential requirements for synthetic bone graft substitutes. However, inorganic composite systems have been assessed and demonstrated to be beneficial for bone tissue regeneration.^7,22^ Recently, Mg alloys or Mg-coated surfaces of metallic substrates have been used in bone tissue engineering due to their in situ degradation, biocompatibility, and mechanical properties to positively stimulate the bone regeneration.^28^ Yoshizawa et al^69^ reported that exposure of Mg ions to human bone marrow stem cells increased matrix production, mineral deposition, and expression of osteoblast genes. Tsai et al^27^ demonstrated that Mg–Ca silicate/PCL 3D scaffolds have the potential to promote osteogenic differentiation of human MSCs, which could enhance the cell adhesion, proliferation, ALP activity, and Ca mineral deposits. Furthermore, it has been found that when MG-63 cells were seeded on hydroxyapatite cement doped with Mg, the alkaline phosphatase activity of cells was increased.^79^ All these exciting results support our findings of better ADSCs adhesion, proliferation, and enhancement of osteogenic markers (ALP activity and Ca deposition) in the case of Mg-filler loaded composite scaffolds.

**Figure 8:**
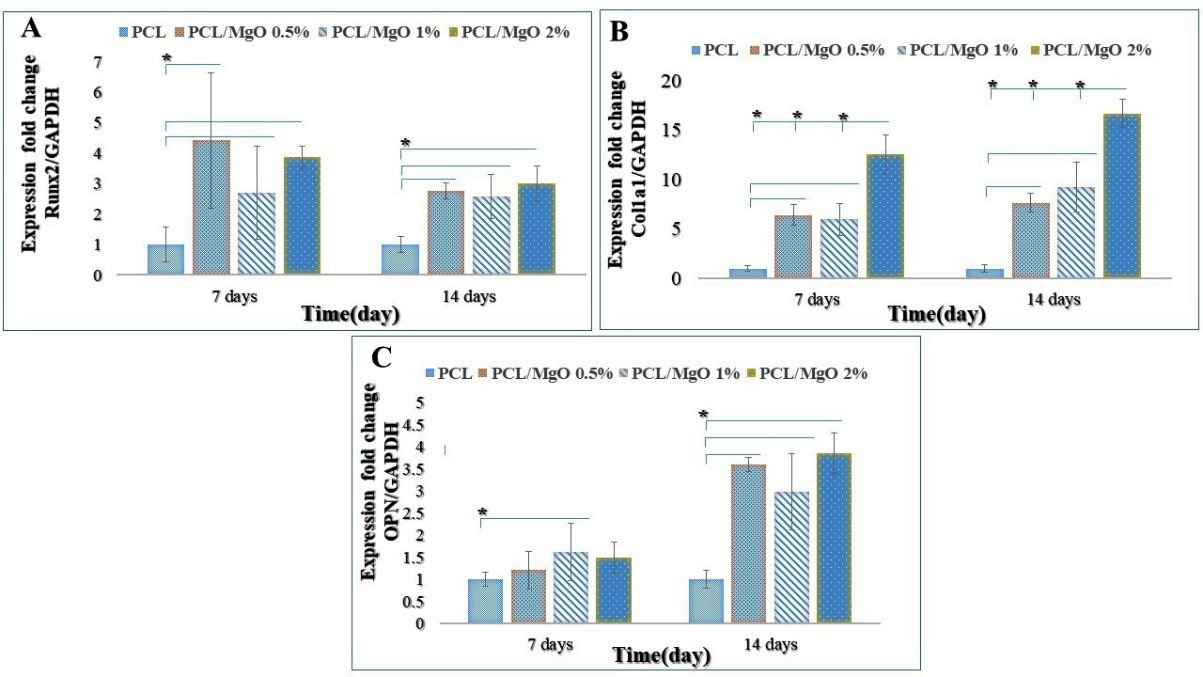
Real-time RT-PCR analysis of cell differentiation on MgO/PCL scaffolds with different MgO concentrations. Relative gene expression of A: Runx2; B: Col1a1; C: OPN after 7 and 14 days (*P < 0.05). As shown, the relative expression of these target genes in ADSCs cultured on MgO/PCL composites was dramatically higher than on pure PCL substrates at 7 and 14 days, except for the expression of OPN on the 7th day. Runx2 expresses during the early stages of differentiation, achieves the highest level in immature osteoblasts, and is down-regulated in mature osteoblasts. The expressions of Col1a1 and OPN were increased, especially in the case of MgO (2%)/PCL, as the culture time was extended (n=3) (*t*tests) (*P < 0.05).

To further investigate the osteogenic differentiation properties, expression of Runx2, Col1a1, and OPN was measured using RT-PCR at 7 and 14 days of culture. Runx2 is a critical transcription factor that regulates osteoblast differentiation and expresses during the early stages of differentiation, achieves the highest level in immature osteoblasts, and down-regulated in mature osteoblasts.^80,81^ Col1a1 protein makes up nearly 90% of bone matrix composition and has a fibrillary structure that creates the foundation of tissue shape by making complex 3D scaffolds and mechanically supports cells. Therefore the expression of Col1a1 is increased during osteoblast differentiation.^82,83^ OPN, as a prominent bone matrix protein, is a marker of the terminal stage of osteoblast differentiation.^84^ As shown in Figure 8, the relative expression of these target genes in ADSCs cultured on MgO/PCL composites was dramatically higher than on pure PCL substrates at 7 and 14 days, except for the expression of OPN on the 7th day. However, the expression of these genes on various composite scaffolds did not exhibit a regular tendency. For Runx2 the expression was dramatically higher on the 0.5- and 2- % MgO/PCL scaffolds on the 7th day. The expression level of Runx2 at the 14^th^ day was decreased in three composite scaffolds rather than that on the 7^th^ day, which could be due to the reality mentioned above that Runx2 is an early marker of osteoblast differentiation, and MgO promotes its expression and maturation at early stages remarkably. As the culture time was extended, the Col1a1 mRNA levels of ADSCs grown on the three composite scaffolds were progressively enhanced. ADSCs subjected to MgO (2%)/PCL nanofibers showed the highest up-regulation in Col1a1 expression on the 7^th^ and 14^th^ day. It is evident from Figure 8C, for OPN there were little differences between the MgO/PCL composite scaffolds and the pure PCL on the 7th day, and its expression is low, which could be because OPN is a late-stage marker of osteoblast differentiation.^84,85^ OPN expressions on 14^th^ day in ADSCs grown on MgO/PCL composite scaffolds were dramatically upregulated compare to pure PCL scaffold, especially the higher expression on the MgO (2%)/PCL scaffold. Cheng et al^86^ reported that open-porous Mg scaffolds have great potential for early vascularization and up-regulation of Col1a1and OPN expression in cells seeded on them, leading to more mature bone formation and higher bone mass. In brief, according to results of cell viability, ALP activity, Ca deposition and RT-PCR assay, we can conclude that the increase of MgO amount in PCL nanofibers (especially 2 wt%) had a significant enhancement for differentiation of the ADSCs into osteoblastic cells. Interestingly, it should be noted that the presence of Mg is crucial for human metabolism and can affect a wide range of cellular functions, including the transportation of Ca^2+^, K^+^, energy metabolism, the modulation of signal transduction pathways, and cell proliferation.^87^ The Mg is well known as biodegradable metal and a critical natural mineral for bone development.^88,89^ Juan et al reported that Mg chloride enhances MSC proliferation by Notch1 signaling activation and induces osteogenic differentiation.^90^ Also, our study further improves that MgO within scaffolds can utilize as a stimulator of osteogenic differentiation and elevating mineralization.

## Conclusion

In this study, MgO/PCL electrospun hybrid scaffolds were fabricated, characterized, and confirmed that the incorporation of the MgO nanoparticles into PCL nanofibers is capable of developing its mechano-chemical properties and biological behavior. In summary, increasing MgO concentration demonstrated a significant effect on reducing fiber diameters. Then the decreased contact angle indicated that the hydrophilic character of PCL had been increased by MgO adding. Boosted bioactivity of the MgO/PCL scaffold was confirmed by enhanced cell adhesion and viability. Moreover, our results demonstrated that MgO/PCL electrospun scaffolds enhanced osteogenic differentiation of ADSCs by showing the significant increase in ALP activity, Ca deposition, and osteogenic-related gene expressions compared to ADSCs on pure PCL electrospun scaffold. According to results are proposed that the MgO/PCL hybrid scaffold could be one of the ideal candidates for osteogenic differentiation of ADSCs and, subsequently, bone tissue engineering.

## Ethical Issues

Ethical approval for this study was given by the Shahid Beheshti University of Medical Sciences (IR.SBMU.REC.1397.047).

## Conflict of Interest

The authors declare that they have no known conflict of interests or personal relationships that could have appeared to influence the research reported in this study.

## Acknowledgments

This work was supported by the Proteomics research center, Shahid Beheshti University of Medical Sciences (Grant No. 15387).
